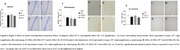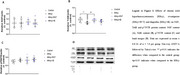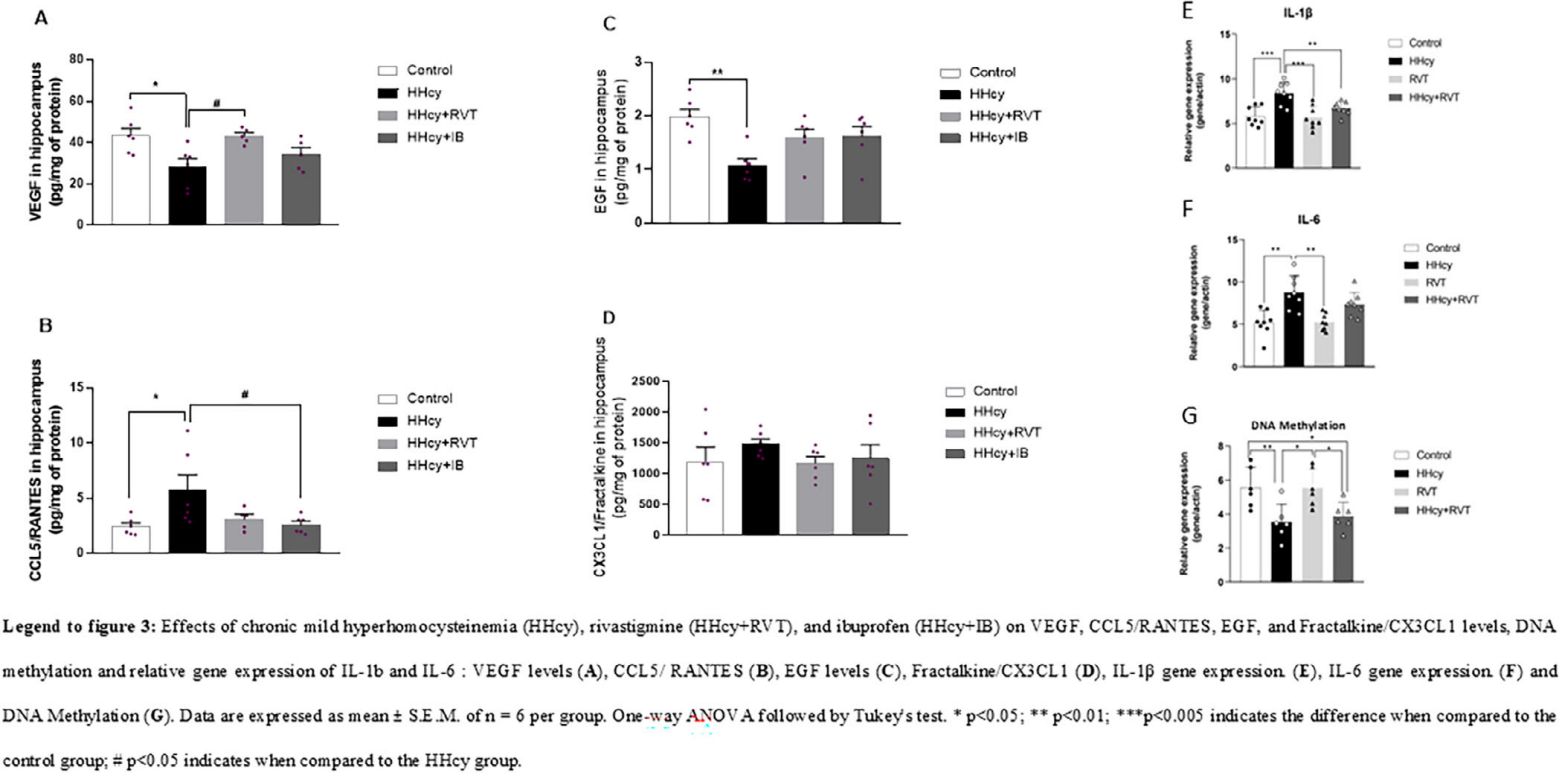# Homocysteine‐Driven Disruption of Neurotrophic and Inflammatory Circuits in the Hippocampus: Novel Insights into Alzheimer's Therapeutics with Rivastigmine and Ibuprofen

**DOI:** 10.1002/alz70861_108978

**Published:** 2025-12-23

**Authors:** Gustavo Ricardo Krupp Prauchner, Osmar Vieira Ramires Junior, Angela Terezinha de Souza Wyse

**Affiliations:** ^1^ Universidade Federal do Rio Grande do Sul, Porto Alegre, Rio Grande do Sul Brazil

## Abstract

**Background:**

Alzheimer's disease (AD) is characterized by cognitive decline, synaptic dysfunction, and neuronal loss, processes linked to neuroinflammation and impaired neurotrophic support. AD research focuses on understanding molecular mechanisms underlying neurodegeneration. Hyperhomocysteinemia (HHcy), characterized by elevated homocysteine levels, has emerged as a significant risk factor for neurodegenerative diseases, including AD. HHcy affects neurotrophic signaling and promotes neuroinflammation. Studies have shown that HHcy mimics several pathological features of AD, including disruption of neurotrophic pathways such as BDNF/TrkB signaling, and induction of chronic glial activation, contributing to synaptic loss and cognitive impairment, making it a valuable model for studying neurodegenerative processes and potential therapeutic interventions.

**Method:**

Mild HHcy was induced in animal models followed by immunohistochemical or biomolecular analysis of hippocampal tissue. The study evaluated glial markers (Iba‐1 and GFAP) (*n* =6), neurotrophic factors (VEGF, EGF, NGF, p75NTR and TrkB) (*n* =6), and inflammatory markers (including CCL5/RANTES, CX3CL1/Fractalkine, IL‐1β and IL‐6) (*n* =6) to assess neuroinflammatory responses. In parallel, DNA methylation profiles were examined to determine epigenetic modifications associated with HHcy. Treatment groups received Rivastigmine or Ibuprofen.

**Result:**

HHcy significantly decreased hippocampal levels of neurotrophic factors VEGF, EGF, and TrkB (*p* <0.05) while increasing the expression of inflammatory markers such as CCL5/RANTES (*p* <0.05) and pro‐inflammatory interleukins Il‐1β and IL‐6 (*p* <0,05). In addition, HHcy decreased DNA methylation levels (*p* <0.05). Rivastigmine treatment reversed the effects of HHcy in TrkB protein content, VEGF levels, relative gene expression of Il‐1β (*p* <0.05) and DNA methylation levels which display a potential neuroprotective effect of the treatment. Also, ibuprofen showed neuroprotective effects lowering CCL5/RANTES content.

**Conclusion:**

HHcy disrupts hippocampal neurotrophic and inflammatory circuits and alters DNA methylation profiles relevant to AD pathology. Rivastigmine treatment reversed alterations in TrkB and VEGF levels, IL‐1β expression, and global DNA methylation, while Ibuprofen reduced CCL5/RANTES content, showing distinct neuroprotective effects through modulation of neurotrophic and inflammatory pathways. These findings highlight the potential of pharmacological strategies targeting key molecular features of AD. Importantly, they reinforce the utility of HHcy as a translational model for AD, providing insight into key processes such as impaired neurotrophic signaling, chronic neuroinflammation, and epigenetic dysregulation ‐ hallmarks of the disease and promising therapeutic targets.